# 3′-UTR engineering to improve soluble expression and fine-tuning of activity of cascade enzymes in *Escherichia coli*

**DOI:** 10.1038/srep29406

**Published:** 2016-07-11

**Authors:** Ji-Won Song, Ji-Min Woo, Gyoo Yeol Jung, Uwe T. Bornscheuer, Jin-Byung Park

**Affiliations:** 1 Department of Food Science & Engineering, Ewha Womans University, Seoul 120-750, Republic of Korea; 2 Department of Chemical Engineering and School of Interdisciplinary Bioscience and Bioengineering, Pohang University of Science and Technology (POSTECH), Gyeongbuk 790-784, Republic of Korea; 3 Institute of Biochemistry, Department of Biotechnology & Enzyme Catalysis, Greifswald University, 17487 Greifswald, Germany

## Abstract

3′-Untranslated region (3′UTR) engineering was investigated to improve solubility of heterologous proteins (e.g., Baeyer-Villiger monooxygenases (BVMOs)) in *Escherichia coli*. Insertion of gene fragments containing putative RNase E recognition sites into the 3′UTR of the BVMO genes led to the reduction of mRNA levels in *E. coli*. Importantly, the amounts of soluble BVMOs were remarkably enhanced resulting in a proportional increase of *in vivo* catalytic activities. Notably, this increase in biocatalytic activity correlated to the number of putative RNase E endonucleolytic cleavage sites in the 3′UTR. For instance, the biotransformation activity of the BVMO BmoF1 (from *Pseudomonas fluorescens* DSM50106) in *E. coli* was linear to the number of RNase E cleavage sites in the 3′UTR. In summary, 3′UTR engineering can be used to improve the soluble expression of heterologous enzymes, thereby fine-tuning the enzyme activity in microbial cells.

Synthetic biology and systems biology allow whole-cell biocatalysis and microbial fermentations to generate a variety of chemical products^1^,^2^,^3^,^4^,^5^,^6^,^7^. Not only small molecules but also large and complex metabolites (e.g., polyphenols, carotenoids, terpenoids, plant oxylipins) could be synthesized from renewable biomass. However, production of these molecules often remains low because of a number of factors including low expression level of the required enzymes in the microbial host. Most of all, oxygenases (e.g., P450 monooxygenases and Baeyer-Villiger monooxygenases (BVMOs)), which serve as one of the key enzymes in preparation of large and complex metabolites (e.g., polyphenols, carotenoids, terpenoids, plant oxylipins) from renewable biomass as well as oxyfunctionalization of hydrocarbons and fatty acids^8^,^9^,^10^, are usually difficult to overexpress in a functional form in bacterial cells^11^,^12^,^13^.

Functional expression of heterologous proteins including oxygenases in bacterial cells can be improved by various methods. Optimization of not only the induction conditions for gene expression (e.g., cultivation temperature, type and concentration of inducer), but also the gene expression systems including the promoters, ribosome binding sites (RBSs), 5′-untranslated region (5′UTR), and codon usage have been largely investigated to enhance soluble expression of enzymes and proteins^14^,^15^,^16^,^17^,^18^. In addition, introduction of molecular chaperones^19^,^20^, the fusion of proteins with soluble peptides and proteins^21^,^22^, and other protein engineering methods (e.g., directed evolution)^23^,^24^ often allowed or improved functional expression of foreign proteins and enzymes in bacterial host cells.

3′UTR has been found to mainly harbor a transcriptional terminator that contributes to RNA stabilization in prokaryotes^25^,^26^. Thereby, it was largely used to increase stability of translationally active mRNAs by inserting the specific sequence (e.g., repetitive extragenic palindromic (REP) sequence) into the 3′UTRs^27^. For example, expression level of the MalE protein in *E. coli* was significantly improved by inserting the REP sequence into its 3′UTR. In this study, 3′UTR engineering was examined as a tool to improve solubility of foreign enzymes in *E. coli*. 3′UTR engineering to reduce the target mRNA level led to significant enhancement of the functional expression of the BVMOs from *Pseudomonas fluorescens* DSM50106^12^ and *Rhodococcus jostii* RHA1^28^, which were otherwise mostly produced in an insoluble form in *E. coli*. The 3′UTR engineering thus also allowed a substantial increase of biotransformation activities of the *E. coli* cells expressing the BVMO enzymes.

## Results

### Functional expression of *bmoF1* in *E. coli*

The BmoF1, which is a Baeyer-Villiger monooxygenase from *P. fluorescens* DSM50106, was reported to catalyze the regiospecific oxygen insertion into the carbon skeletons adjacent to a keto-group of various compounds of interest^12^. However, it was very difficult to express soluble enzyme in *E. coli* strains (Fig. S1). Not only engineering of gene expression systems and expression conditions, but also the introduction of molecular chaperones and directed evolution of the *bmoF1* did not allow satisfactory functional expression in *E. coli* cells^12^,^24^. 5′UTR engineering, which was used to regulate expression level of the soluble proteins (e.g., green fluorescent protein^16^, phosphoenolpyruvate synthase^29^, γ-prefoldin and recombinant thermosome^20^, was also not so useful to improve soluble expression of the insoluble enzyme (data not shown).

**Figure 1:**
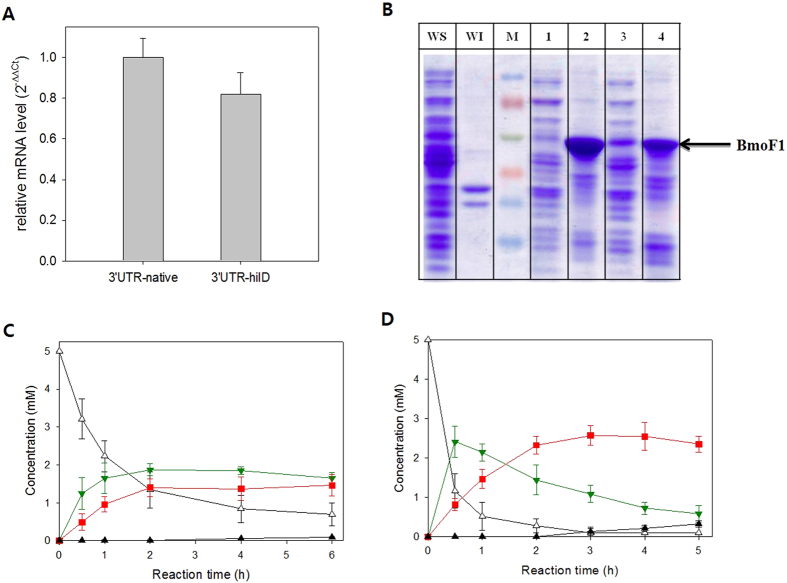
(**A**) mRNA level of *bmoF1*-3′UTR_native_ (3′UTR-native) and *bmoF1*-3′UTR_*hilD*_ (3′UTR-hilD) in *E. coli* BL21(DE3). The samples were prepared from cultures of the recombinant *E. coli* BL21(DE3) pET22b-BmoF1-3′UTR_native_ and *E. coli* BL21(DE3) pET22b-BmoF1-3′UTR_*hilD*_. The mRNA level of *ihfB* was used for normalization. The relative mRNA level was estimated by ΔΔCt method. (**B**) SDS-PAGE analysis of cell extracts from the wild type *E. coli* BL21(DE3) (lane WS and WI), the recombinant *E. coli* BL21(DE3) pET22b-BmoF1-3′UTR_native_ (lanes 1 and 2), and *E. coli* BL21(DE3) pET22b-BmoF1-3′UTR_*hilD*_ (lanes 3 and 4). Lane M, Marker; lane WS, 1, and 3, soluble fraction; lane WI, 2 and 4, insoluble fraction. (**C**) Biotransformation of ricinoleic acid (**1**) into ester **3** by the recombinant *E. coli* BL21(DE3) pCOLA-ADH, pET22b-BmoF1-3′UTR_native_ and (**D**) *E. coli* BL21(DE3) pCOLA-ADH, pET22b-BmoF1-3′UTR_*hilD*_. The experiments were carried out in triplicate and the error bars indicate standard deviation. The symbols indicate concentrations of ricinoleic acid (△), 12-ketooleic acid (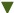), ester (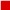) and 11-hydroxyundec-9-enoic acid (▲).

According to a recent study, stability of *Salmonella enterica hilD* mRNA was markedly influenced by the presence or absence of its 3′UTR, which consists of 310 nucleotides including fourteen putative RNase E cleavage sites (Fig. S2A)^30^. For instance, deletion of the 3′UTR led to an increase of *hilD* mRNA level and thus enhancement of the gene expression. Based on this study, we assumed that stability of the *bmoF1* mRNA could be modulated via engineering 3′UTR, which may influence local concentration of the newly synthesized BmoF1 polypeptides in the cytoplasm. Firstly, the *hilD* 3′UTR was introduced to serve as the 3′UTR of *bmoF1* (see the Materials and Methods for details). After construction of *E. coli* BL21(DE3) pET-BmoF1-3′UTR_*hilD*_, the *bmoF1* mRNA level was compared with *E. coli* BL21(DE3) pET-BmoF1-3′UTR_native_ (Figs 1A and S3A). The mRNA level of *bmoF1*-3′UTR_*hilD*_ was slightly lower than the *bmoF1*-3′UTR_native_, suggesting that 3′UTR_*hilD*_ in *E. coli* appears to influence stability of the mRNAs.

We have next examined expression level of the *bmoF1*-3′UTR_*hilD*_ by SDS-PAGE analysis. The total expression level of *bmoF1*-3′UTR_*hilD*_ was lower than *bmoF1*- 3′UTR_native_ (Fig. 1B), whereas the soluble expression level was markedly greater in *bmoF1*- 3′UTR_*hilD*_. This result indicated that soluble expression of *bmoF1* could be improved via decreasing stability of its mRNAs.

The BmoF1 was reported to be too unstable *in vitro* to be isolated in a pure form^12^,^21^. Thereby, the catalytic activity of BmoF1 was examined by whole-cell biotransformations. The recombinant *E. coli* BL21(DE3) pCOLA-ADH and pET22b-BmoF1-3′UTR_*hilD*_, expressing the long-chain secondary alcohol dehydrogenase (ADH) of *M. luteus* NCTC2665, which generates the substrates of BmoF1 (Scheme S1), in addition to BmoF1, was constructed. When ricinoleic acid was added into the reaction medium containing *E. coli* pCOLA-ADH and pET22b-BmoF1-3′UTR_*hilD*_, the BmoF1 reaction product (**3**) was produced at a specific formation rate and bioconversion yield, which were significantly higher than using the recombinant *E. coli* pCOLA-ADH and pET22b-BmoF1 with 3′UTR_native_ (Fig. 1C,D). These results suggested that insertion of the 3′UTR_*hilD*_ sequence might result in a decrease of the stability of the *bmoF1* mRNA, which might in turn reduce the BmoF1 expression level, but overall improve the gene expression in a functional form in *E. coli* BL21(DE3).

### Effect of 3′UTR_CAT_ on expression of the *bmoF1*

**Figure 2:**
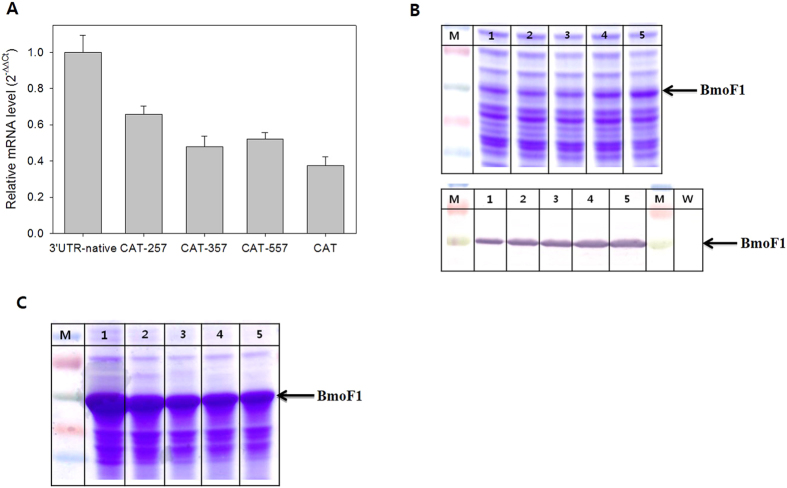
(**A**) mRNA level of *bmoF1* with 3′UTR_CAT_ and its variants. The samples were prepared from cultures of the recombinant *E. coli* BL21(DE3) pET22b-BmoF1-3′ UTR_native_ (3′UTR-native), *E. coli* BL21(DE3) pET22b-BmoF1-3′ UTR_CAT-257_ (CAT-257), *E. coli* BL21(DE3) pET22b-BmoF1-3′ UTR_CAT-357_ (CAT-357), *E. coli* BL21(DE3) pET22b-BmoF1-3′ UTR_CAT-557_ (CAT-557), and *E. coli* BL21(DE3) pET22b-BmoF1-3′ UTR_CAT-657_ (CAT). The mRNA level of *ihfB* was used for normalization. The relative mRNA level was estimated by the ΔΔCt method (see Materials and Methods for details). (**B**) SDS-PAGE (upper panel) and Western Blot (lower panel) analysis of soluble fraction of cell extracts prepared from culture of the recombinant *E. coli* BL21(DE3) pET22b-BmoF1-3′ UTR_native_ (lane 1), *E. coli* BL21(DE3) pET22b-BmoF1-3′ UTR_CAT-257_ (lane 2), *E. coli* BL21(DE3) pET22b-BmoF1-3′ UTR_CAT-357_ (lane 3), *E. coli* BL21(DE3) pET22b-BmoF1-3′ UTR_CAT-557_ (lane 4), and *E. coli* BL21(DE3) pET22b-BmoF1-3′ UTR_CAT-657_ (lane 5). Lane M, Marker. (**C**) SDS-PAGE analysis of insoluble fraction of cell extracts prepared from cultures of the recombinant *E. coli* BL21(DE3) pET22b-BmoF1-3′ UTR_native_ (lane 1), *E. coli* BL21(DE3) pET22b-BmoF1-3′ UTR_CAT-257_ (lane 2), *E. coli* BL21(DE3) pET22b-BmoF1-3′ UTR_CAT-357_ (lane 3), *E. coli* BL21(DE3) pET22b-BmoF1-3′ UTR_CAT-557_ (lane 4), and *E. coli* BL21(DE3) pET22b-BmoF1-3′ UTR_CAT-657_ (lane 5). Lane M, Marker.

With an aim to further investigate the impact of 3′UTR engineering, we examined the CAT sequence as an alternative 3′UTR. The CAT sequence, which encodes the chloramphenicol acetyltransferase of *E. coli*, contains twenty eight putative RNase E recognition sites^31^,^32^ (Fig. S2B). After construction of *E. coli* BL21(DE3) pET22b-BmoF1-3′UTR_CAT_ to contain the full-length CAT coding sequence in the 3′UTR, the *bmoF1* mRNA level was compared with *E. coli* BL21(DE3) pET22b-BmoF1 with 3′UTR_native_, which contains 5 putative RNase E recognition sites, by not only RT-PCR (Fig. S3B) but also qRT-PCR (Fig. 2A). The mRNA level of the *bmoF1*-3′UTR_CAT_ was about 2.6-fold lower, compared to the *bmoF1*-3′UTR_native_, indicating that the CAT sequence can be used as an active 3′UTR. The SDS-PAGE and Western Blot analysis of BmoF1 also demonstrated that the total expression level of *bmoF1*-3′UTR_CAT_ was lower than *bmoF1*-3′UTR_native_, whereas the soluble expression level was markedly greater in *bmoF1*- 3′UTR_CAT_ (Fig. 2B,C).

To test whether the number of RNase E cleavage sites in the CAT sequence correlates with mRNA stability, we constructed a set of variants containing partial deletions of the 3′UTR_CAT_. The 3′UTR variants, which contained 257, 357 and 557 nucleotides from 5′ region of the CAT sequence, showed a step by step increase of the number of putative RNase E recognition sites (Fig. S2B,C). Notably, as the length of the 3′UTR_CAT_ increased from 257 to 657 nucleotides, the *bmoF1* mRNA level was gradually reduced (Figs 2A and S3B). The SDS-PAGE and Western Blot analysis of BmoF1s also showed a correlation between length of 3′UTR (number of RNase E cleavage sites) and soluble expression level in the cytoplasm (Figs 2B,C and S4). These results indicated that the CAT coding region really served as 3′UTR of *bmoF1*, which has an influence on the stability of the *bmoF1* mRNAs and thus on the protein expression level.

### Relationship between number of RNase E cleavage sites and biocatalytic activity

**Figure 3:**
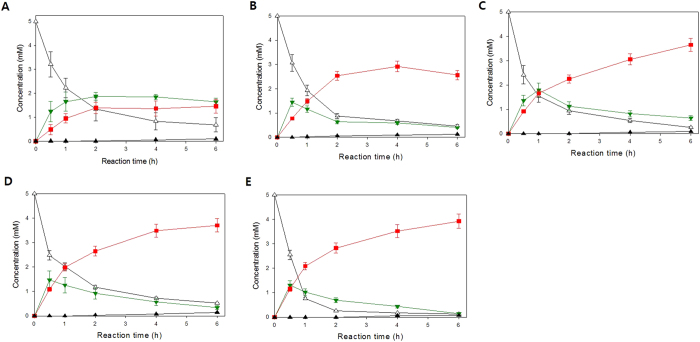
Biotransformation of ricinoleic acid (**1**) into ester **3** by (**A**) the recombinant *E. coli* BL21(DE3) pCOLA-ADH, pET22b-BmoF1-3′ UTR_native_, (**B**) *E. coli* BL21(DE3) pCOLA-ADH, pET22b-BmoF1-3′ UTR_CAT-257_, (**C**) *E. coli* BL21(DE3) pCOLA-ADH, pET22b-BmoF1-3′ UTR_CAT-357_, (**D**) *E. coli* BL21(DE3) pCOLA-ADH, pET22b-BmoF1-3′ UTR_CAT-557_, and (**E**) *E. coli* BL21(DE3) pCOLA-ADH, pET22b-BmoF1-3′ UTR_CAT-657_. The experiments were carried out in triplicate and the error bars indicate standard deviation. The symbols indicate concentrations of ricinoleic acid (△), 12-ketooleic acid (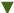), ester (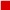) and 11-hydroxyundec-9-enoic acid (▲).

**Figure 4:**
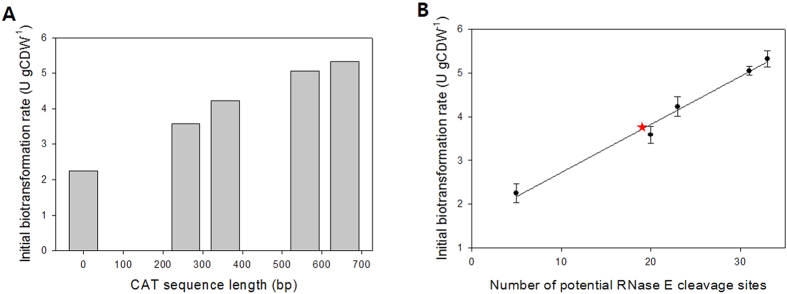
(**A**) Relationship between length of 3′UTR_CAT_ and initial biotransformation rate, which was calculated based on the product concentration measured at t = 0.5 h by GC/MS (Fig. 3). Zero in the X-axis indicated the initial biotransformation rate of the recombinant *E. coli* BL21(DE3) pCOLA-ADH, pET22b-BmoF1-3′UTR_native_, containing 5 potential RNase E cleavage sites. (**B**) Relationship between the number of potential RNase E cleavage sites in the 3′UTR_CAT_ and initial biotransformation rate. Five in the X-axis indicated the initial biotransformation rate of the recombinant *E. coli* BL21(DE3) pCOLA-ADH, pET22b-BmoF1-3′UTR_native_, containing 5 potential RNase E cleavage sites. The red star indicates the initial biotransformation rate of the recombinant *E. coli* BL21(DE3) pCOLA-ADH, pET22b-BmoF1-3′UTR_*hilD*_, containing 19 potential RNase E cleavage sites.

To investigate the effect of the number of RNase E cleavage sites of 3′UTR on biocatalytic activity of BmoF1 in *E. coli*, whole cell biotransformation of ricinoleic acid was carried out with the recombinant *E. coli* BL21(DE3) expressing the ADH and BmoF1 with 3′UTR_native_ or 3′UTR_CAT_ variants. The reaction conditions were the same as those shown in Fig. 1C. The ricinoleic acid biotransformation activity of the recombinant *E. coli* BL21 (DE3) pCOLA-ADH, pET22b-BmoF1-3′UTR_CAT257_, -3′UTR_CAT357,_ -3′UTR_CAT557_, and -3′UTR_CAT_ showed a stepwise increase in the biotransformation activities and product yields, compared to the BmoF1-3′UTR_native_ (Fig. 3 and Table 1). Also, there was an excellent correlation between number of RNase E sites and the initial biotransformation rates, which were measured on the basis of the ester (**3**) concentrations at t = 0.5 h (Fig. 4). The initial biotransformation rate of recombinant *E. coli* BL21(DE3) pCOLA-ADH and pET22b-BmoF1-3′UTR_*hilD*_ (i.e., red star in Fig. 4B) also correlated with the number of RNase E sites. The results indicated that the number of RNase E sites rather than the 3′UTR sequence is critical for the gene expression and hence biotransformation rates. Overall, it was concluded that the functional expression level of the *bmoF1* was dependent upon number of RNase E cleavage sites in its 3′UTR.

### Soluble expression of a BVMO from *Rhodococcus jostii* RHA1

**Figure 5:**
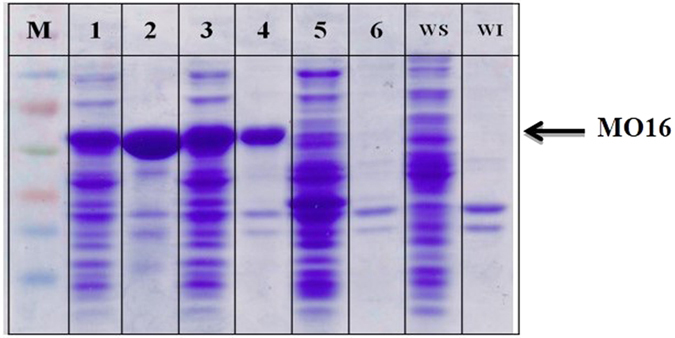
The SDS-PAGE analysis of cell extracts prepared from culture of *E. coli* BL21(DE3) (lane WS and WI), the recombinant *E. coli* BL21(DE3) pET-MO16-3′UTR_native_ (lanes 1 and 2), *E. coli* BL21(DE3) pET-MO16-3′UTR_CAT-257_ (lanes 3 and 4), and *E. coli* BL21(DE3) pET-MO16-3′UTR_CAT-657_ (lanes 5 and 6). Lanes WS, 1, 3, and 5 indicate soluble fraction of cell extracts. Lanes WI, 2, 4, and 6 indicate insoluble fraction of cell extracts. Lane M, Marker.

**Figure 6:**
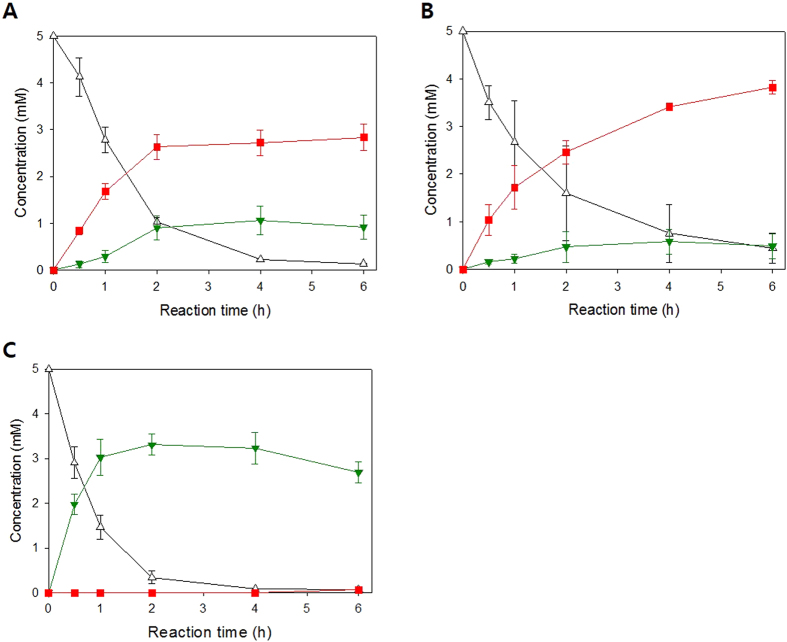
Biotransformation of ricinoleic acid (**1**) into ester **3** by (**A**) the recombinant *E. coli* BL21(DE3) pCOLA-ADH, pET-MO16-3′UTR_native_, (**B**) *E. coli* BL21(DE3) pCOLA-ADH, pET-MO16-3′UTR_CAT-257_, and (**C**) *E. coli* BL21(DE3) pCOLA-ADH, pET-MO16-3′UTR_CAT-657_. The experiments were carried out in triplicate and the error bars indicate standard deviation. The symbols indicate concentrations of ricinoleic acid (△), 12-ketooleic acid (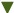), ester (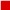) and 11-hydroxyundec-9-enoic acid (▲).

The BVMO from *R. jostii* RHA1 (i.e., MO16, sequence identity with BmoF1: 23.3%^28^) is another protein, which remained very difficult to express in a functional form in *E. coli* cells (Fig. S5). The enzyme was mostly produced as inclusion body in *E. coli* BL21 (DE3). Thereby, we have examined effect of 3′UTR on expression of the enzyme in *E. coli*. The MO16-3′UTR_CAT-657_ and MO16-3′UTR_CAT-257_ variants were constructed and subjected to SDS-PAGE analysis (Fig. 5) and whole-cell biotransformation of ricinoleic acid (Fig. 6). Surprisingly, introduction of the 3′UTR_CAT-657_ did not allow any expression in *E. coli*; the protein band was rarely detectable in the SDS-PAGE analysis (Fig. 5). The accumulation of the 12-ketooleic acid (**2**) without formation of the MO16 reaction product (**3**) (Scheme S1) (Fig. 6) in the culture medium confirmed that the enzyme was hardly expressed in *E. coli*. In contrast, the MO16 enzyme from the MO16-3′UTR_CAT-257_ variant was produced rather in a soluble form (Fig. 5). The increase of soluble protein led to formation of the ester product (**3**) to a rate, which is ca. 35% greater than the MO16-3′UTR_native_ (Fig. 6). The same trend was also observed in the biotransformation of 13-hydroxyoleic acid (Fig. S6) (Scheme S2). These results indicated that 3′UTR engineering could be used to improve soluble expression of the enzymes in *E. coli* as well as to fine-tune the enzyme activity in microbial cells.

## Discussion

Expression of enzymes and proteins in a functional form in microbial cells is a key issue in metabolic engineering as well as in whole-cell biocatalysis. There have been a number of approaches to improve soluble expression of enzymes and proteins (see the reviews by Mahalik *et al*.^14^ and Rosano and Ceccarelli (2014) for details^14^,^15^). Here, we described a novel method to enhance functional expression of recombinant enzymes as exemplified for two monooxygenases (e.g., BmoF1 from *P. fluorescens* and MO16 from *R. jostii*). Depending on the number of putative RNase E endonucleolytic cleavage sites in the 3′UTR, expression level of the BmoF1 and MO16 in a soluble form as well as *in vivo* catalytic activity were largely influenced. Especially, expression of the MO16 was much more responsive to the number of RNase E cleavage sites in the 3′UTR (Figs 5 and 6); the MO16-3′UTR_native_ (total No. of RNase E cleavage sites: 10) resulted in the expression of mostly insoluble protein, whereas the MO16-3′UTR_CAT-257_ (total No. of RNase E cleavage sites: 25) led to better expression in soluble form (Fig. 5). The MO16-3′UTR_CAT-657_ (total No. of RNase E cleavage sites: 33) did not allow any expression in *E. coli* BL21(DE3). Whole-cell biotransformation demonstrated that the amount of functional MO16 from the MO16-3′UTR_CAT-257_ was significantly greater than the MO16-3′UTR_native_ or the MO16-3′UTR_CAT-657_ (Fig. 6).

Although the mechanism(s) remained to be investigated, the smaller amount of mRNAs from the genes having the 3′UTR_*hilD*_ or the 3′UTR_CAT_ suggested that the number of the RNase E cleavage sites in the 3′UTR might affect stability of the corresponding mRNAs. This assumption is also supported by previous studies, which showed that degradation of certain bacterial RNAs might involve endonucleolytic cleavages by RNase E at a single-stranded AU-rich region in the 3′UTR^30^,^33^,^34^. In *E. coli*, the degradation of many mRNAs is initiated by an endonucleolytic cleavage catalyzed by RNase E, which products serve as the substrates for additional RNase E cleavages as well as for digestion by the 3′→5′exonucleases, polynucleotide phosphorylase (PNPase) and RNase II^35^,^36^,^37^,^38^. Figure S4 indicates that the low mRNA stability seemed to result in reduction of the *bmoF1* mRNA level in *E. coli* BL21(DE3), which in turn decreased total expression level of *bmoF1*. The impact of mRNA stability on the protein expression level was more clearly shown in engineering of 3′UTR of MO16 (Fig. 5). The increase in the number of RNase E cleavage sites in the 3′UTR by inserting the CAT sequence did not allow any expression of the MO16 enzyme in *E. coli* BL21(DE3). Therefore, it was assumed that 3′UTR engineering is a powerful tool to regulate total protein expression level in *E. coli*.

The low mRNA stability might also cause a decrease in the number of the target polypeptides produced per mRNA, which may in turn lead to low local concentrations of the polypeptides or unfolded proteins (Fig. S4). These low local concentrations might reduce the possibility of inclusion body formation of the unfolded or partially folded proteins. This would be one of the major differences as compared to other methods based on regulation of transcription rates (e.g., by promoter engineering). Although the transcription rate of *bmoF1* was markedly reduced by changing the strong T7 promoter to the mild rhamnose promoter, the amount of BmoF1 in a functional form remained unchanged^12^. Thereby, it was assumed that one of the mechanisms of 3′UTR engineering might include reduction of the number of the target polypeptides produced per mRNA.

In view of synthetic biology and metabolic engineering, functional gene expression and regulation of the enzyme activities involved in biocatalysis or biosynthesis play key roles to achieve high productivities and product yields. The stepwise increase of soluble expression level and *in vivo* catalytic activity of the BVMO enzymes depending on number of RNase E cleavage sites in the 3′UTR points out that 3′UTR engineering could be used as one of the tools to achieve fine-tuning of the catalytic enzyme activities in whole-cell biocatalysis and microbial fermentations. Obviously, a balance must be found for the optimal concentration of the mRNA encoding the enzyme of interest.

BVMOs are oxidative enzymes that catalyze the Baeyer–Villiger oxidations and sulfoxidations with high chemo-, regio-, and enantioselectivity^39^,^40^,^41^,^42^,^43^. Since a variety of aldehydes and (a) cyclic ketones could be converted into their corresponding esters and lactones, BVMOs are interesting candidates for various synthetic applications. For instance, BmoF1 from *P. fluorescens* is able to catalyze regiospecific oxygenation of 12-keto-*cis*-9-octadecenoic acid, 13-keto-*cis*-9-octadecenoic acid, 10-keto-octadecanoic acid, 10-keto-*cis*-12-octadecenoic acid, 13-keto-*cis*-6,9-octadecadienoic acid, and 10-keto-*cis*-6,12-octadecadienoic acid into the corresponding esters^10^,^44^,^45^,^46^,^47^. However, a number of BVMO encoding genes including *bmoF1* and MO16 were difficult to be expressed in a functional form in conventional microbial biocatalysts (e.g., *E. coli*, *Saccharomyces cerevisiae*)^12^,^13^,^48^. Here, functional expression of one of the most insoluble enzymes (i.e., BmoF1 and MO16) was significantly improved via 3′UTR engineering. Therefore, this study will contribute to development of oxygenase-based whole-cell biocatalysis.

In summary, this study demonstrated that 3′-UTR engineering can be used to improve soluble expression and fine-tuning of activity of the catalytic enzymes, which is essential for metabolic engineering for production of value-added chemical products as well as for whole-cell biocatalysis.

## Materials and Methods

### Microbial strains and culture media

Recombinant *E. coli* BL21(DE3) strains were cultivated in Lysogeny broth (5 g L^−1^ yeast extract, 10 g L^−1^ tryptone, and 10 g L^−1^ NaCl) supplemented with 50 μg mL^−1^ of kanamycin and 100 μg mL^−1^ of ampicillin to prepare the plasmid DNAs and cultivation seeds. Riesenberg medium^49^ supplemented with 10 g L^−1^ glucose, 50 μg mL^−1^ kanamycin and 100 μg mL^−1^ ampicillin was used for cultivation and biotransformation. The Riesenberg medium consisted of 4 g L^−1^ (NH_4_)_2_HPO_4_, 13.5 g L^−1^ KH_2_PO_4_, 1.7 g L^−1^ citric acid, 1.4 g L^−1^ MgSO_4_, and 10 mL L^−1^ trace metal solution (10 g L^−1^ FeSO_4_, 2.25 g L^−1^ ZnSO_4_, 1.0 g L^−1^ CuSO_4_, 0.5 g L^−1^ MnSO_4_, 0.23 g L^−1^ Na_2_B_4_O_7_, 2.0 g L^−1^ CaCl_2_, and 0.1 g L^−1^ (NH_4_)_6_Mo_7_O_24_). Expression of the recombinant genes was induced by adding 0.1 mM isopropyl-β-D-1-thiogalactopyranoside (IPTG) to the culture broth at an optical density (OD600) of 0.6 followed by 20 h incubation at 20 °C.

### Gene cloning

3′UTR of *Salmonella enterica hilD* mRNA (3′UTR_*hilD*_) (GenBank accession code: CP012681.1) was synthesized by Cosmogene Tech (Seoul, Korea) and amplified via polymerase chain reaction (PCR) with *hilD*_F and *hilD*_R as the forward and reverse primers (See the Supporting Information, Table S1). The CAT sequence of *E. coli* was PCR-amplified from the pACYCDuet-1 vector with CAT_F and CAT_R. These fragments were subcloned into the HindIII-XhoI site of the pET22b-BmoF1 expressing a BVMO of *P. fluorescens*^12^ resulting in the pET22b-BmoF1-3′UTR_*hilD*_ and pET22b-BmoF1-3′UTR_CAT_ (Table S2), respectively. To construct partial deletion alleles of 3′UTR_CAT_, CAT_F as the forward primer and three different reverse primers (CAT257_R, CAT357_R, CAT557_R) were used for amplification and were also inserted into pET22b-BmoF1.

To construct pET-MO16-3′UTR_CAT-657_ and MO16-3′UTR_CAT-257_, the MO16 gene (GenBank accession code: ABG94724.1) of *Rhodococcus jostii* RHA1^28^ was amplified from pET-MO16 via PCR with MO16_F and MO16_R as the forward and reverse primers. The CAT sequence was amplified from the pACYCDuet-1 vector with MO16_CAT_F and MO16_CAT_R or MO16_CAT257_R. These fragments were subcloned into the HindIII-XhoI site of the pET-MO16 resulting in the pET-MO16-3′UTR_CAT_ and pET-MO16-3′UTR_CAT_, respectively. The sequences of all the constructed plasmids were confirmed by Macrogen (Seoul, Korea).

### RNA extraction and reverse transcription PCR (RT-PCR)

To extract total RNA from the recombinant *E. coli* BL21(DE3) pCOLA-ADH, pET22b-BmoF1with 3′UTR_native_ or 3′UTR_CAT_ variants, the cells were cultivated in Riesenberg medium at 30 °C, and expression of the target genes was induced with 0.1 mM IPTG at an OD_600_ of 0.6. Thereafter, the cultivation temperature was reduced to 20 °C. When the culture reached the stationary growth phase, a 500 μL from a culture broth of cells at stationary phase was centrifuged at 17,230 g for 5 min at 4 °C. Total RNA was extracted by using an RNeasy extraction kit (Qiagen) according to the manufacturer’s instructions. The extracted RNA concentration was determined by using a nano-spectrophotometer.

RT-PCR was carried out to estimate the mRNA expression level of *bmoF1* with HiPi™ One-step 5× RT-PCR premix according to the manufacturer’s instructions. 10 nmol of the extracted RNA and the forward and reverse primers (5 pmol) were mixed with the RT-PCR premix and RNase-free water was added up to 20 μL. The RT-PCR steps were as follows: cDNA was synthesized at 42 °C for 30 min and at 94 °C for 5 min. Amplification step was 35 cycles of 94 °C for 30 s, 60 °C for 30 s, and 72 °C for 45 s. The primer sequences for amplification of *bmoF1* were 5′-ATGAATGCCCACAGTGATTCCATCGACATCGCCATCATCGGTTCGGGT TTTGCC G-3′ (forward primer) and 5′-TGAGAGGCTGCCTTCTGCCGTGGAGTGGGGCGCCGTGGCCGGACGGGGCGGCGC A-3′ (reverse primer). The PCR primer sequences for amplification of *ihfB*^50^ gene for normalization were 5′-ATGACCAAGTCAGAATTGATAGAAAGACTTGCCACCCAGCAATCGCACATTCCCG-3′ (forward primer) and 5′-TTAACCGTAAATATTGGCGCGATCGCGCAGTTCTTTACCAGGTTTAAAGTGAGGA-3′ (reverse primer). The gel electrophoresis was conducted for analysis of RT-PCR products. Relative quantification of the *bmoF1* mRNA level was performed by densitometry (ImageJ software; NIH), normalized against the mRNA level of *ihfB* and calibrated with a *bmoF1* with 3′UTR_native_.

### Quantitative Real Time-PCR (qRT-PCR)

qRT-PCR analysis was conducted to quantify the RNA level. First-strand cDNAs were obtained by reverse transcription of 1 μg of total RNA using SuperScriptTM II Reverse Transcriptase (Invitrogen) according to the manufacturer’s protocol. Real-time gene expression analysis of target gene (*bmoF1*) was performed using StepOnePlus Real-Time PCR System (Applied Biosystems) with the integration host factor subunit beta gene (*ihfB*) as the reference. The Real-Time PCR primer sequences for amplification of the *bmoF1* gene were 5′-CCCAACCTGTTTCTGATCAT-3′ (forward primer) and 5′-GCCTCGATCATCAGGATCAT-3′ (reverse primer). The Real-Time PCR primer sequences for amplification of the *ihfB*^50^ gene for normalization were 5′-TATTGAAATCCGCGGTTTCG-3′(forward primer) and 5′-TCGCCAGTCTTCGGATTACG-3′ (reverse primer). The Real-Time PCR was performed in 50 μL reaction volumes that contained 2x Power SYBR Green PCR Master Mix (Applied Biosystems) and 1 mM forward and reverse primers. The qRT-PCR conditions were as follows: 10 min at 95 °C followed by 40 cycles consisting of 15 s at 95 °C and 30 s at 59 °C. Ct method was applied in relative mRNA quantification, according to the ΔΔCt method^51^. ΔCt was calculated by a Ct value of *bmoF1*taking away that of *ihfB* and three ΔCt was defined as amplified. The mRNA level of *bmoF1* with 3′UTR_native_ was used as control.

### SDS-PAGE and Western Blot analysis

The soluble fraction and insoluble fractions of whole cell extracts were prepared by sonication and centrifugation at 17,230 g for 10 min at 4 °C. The soluble and insoluble fractions were loaded and run onto 10% sodium dodecyl sulfatepolyacrylamide gels, and the separated proteins were confirmed by staining with Coomassie Brilliant Blue R-250.

Western blot analysis was conducted as previously reported^20^. The seperated proteins by SDS-PAGE were transferred to polyvinylidene difluoride (PVDF) membranes. The membranes were placed overnight in PBS containing 5% skim milk and 0.5% Tween 20 to prevent non-specific binding of the membrane with the antibody. After blocking, the membranes were incubated with dilute solution of anti-His primary antibodies (IG Therapy Co., Ltd. Tokyo, Japan) in PBS containing 0.5% Tween 20 and 5% skim milk at a 1:5,000 dilution for 1 h at room temperature. To remove non-specific binding, the membranes was washed by PBS containing 0.5% Tween 20, and then incubated for 1 h at room temperature with dilute solution of goat anti-mouse IgG conjugated to alkaline phosphatase (Abcam, Cambridge, UK) in PBS containing 0.5% Tween 20 and 5% skim milk at a 1:5,000. Development of the blot was conducted with the BCIP/NBT chromogenic solution substrate (Abcam).

Relative quantification of the total and soluble protein expression level was performed by densitometry (ImageJ software; NIH) and calibrated with the total and soluble protein expression level in recombinant *E. coli* BL21(DE3) expressing the *bmoF1* with 3′UTR_native_.

### Whole cell biotransformation

Biotransformation of ricinoleic acid with recombinant *E. coli* was carried out on the basis of our earlier work^45^. Briefly, the recombinant cells were cultivated in Riesenberg medium at 30 °C, and expression of the target genes was induced with 0.1 mM IPTG at an OD_600_ of 0.6. Thereafter, the cultivation temperature was reduced to 20 °C. When the culture reached the stationary growth phase, the recombinant *E. coli* BL21(DE3) pCOLA-ADH, pET22b-BmoF1, which expresses the alcohol dehydrogenase (ADH) of *M. luteus* NCTC2665 and the BVMO of *P. fluorescens* DSM 50106, was harvested by centrifugation at 3,500 g for 20 min at 4 °C and resuspended to 7.2 g dry cells L^−1^ into 50 mM Tris-HCl buffer (pH 8.0). The biotransformation was initiated by adding 5 mM ricinoleic acid and 0.5 g L^−1^ Tween 80 into the 50 mM Tris-HCl buffer (pH 8.0). The biotransformations were conducted at 35 °C and 200 rpm in shaking incubator.

In case of biotransformation with *E. coli* BL21(DE3) pCOLA-ADH, pET-MO16, which expresses the ADH of *M. luteus* NCTC2665 and the BVMO of *R. jostii* RHA1, the biotransformation was initiated at the stationary growth phase (cell concentration: 3.0 g dry cells L^−1^) by adding 5 mM ricinoleic acid or 13-hydroxyoleic acid and 0.5 g L^−1^ Tween 80 into the culture broth on a basis of our previous study^52^.

### Product analysis by GC/MS

Concentrations of remaining ricinoleic acid and accumulating fatty acid products in the medium (i.e., ketone **2** and ester **3**) were determined as described previously^45^. The reaction medium was mixed with a 3-fold volume of ethyl acetate containing 5 g L^−1^ palmitic acid as an internal standard. The organic phase was harvested after vigorous vortexing and then subjected to derivatization with N-methyl- N-(trimethylsilyl)trifluoroacetamide (TMS). The TMS derivatives were analyzed using a Thermo Ultra Trace GC system connected to an ion trap mass detector (Thermo ITQ1100 GC-ion Trap MS, Thermo Scientific). The derivatives were separated on a nonpolar capillary column (30-m length, 0.25-μm film thickness, HP-5MS, Agilent). A linear temperature gradient was programmed as follows: 90 °C, 5 °C min^−1^ to 280 °C. The injection port temperature was 230 °C. Mass spectra were obtained by electron impact ionization at 70 eV. Scan spectra were obtained within the range of 100–600 m/z. Selected ion monitoring (SIM) was used for the detection and fragmentation analysis of the reaction products. Concentration of the reaction substrates and products was determined on a basis of calibration curves, which were determined using commercially available products or products isolated in our lab^45^.

## Additional Information

**How to cite this article**: Song, J.-W. *et al*. 3′-UTR engineering to improve soluble expression and fine-tuning of activity of cascade enzymes in *Escherichia coli*. *Sci. Rep*. **6**, 29406; doi: 10.1038/srep29406 (2016).

## Supplementary Material

Supplementary Information

## Figures and Tables

**Table 1:** Effect of 3′UTR on expression and biotransformation activity of BmoF1.

	Relative mRNA level^a^	Initial biotransformation rates (U g CDW^−1^)^b^	Product yield (%)^c^
*bmoF1*-3′UTR_native_	1.0	2.26 ± 0.31	29.2 ± 0.28
*bmoF1*-3′UTR_CAT-257_	0.66	3.59 ± 0.27	51.3 ± 0.19
*bmoF1*-3′UTR_CAT-357_	0.48	4.23 ± 0.31	72.9 ± 0.27
*bmoF1*-3′UTR_CAT-557_	0.52	5.05 ± 0.15	74.0 ± 0.28
*bmoF1*-3′UTR_CAT-657_	0.37	5.32 ± 0.26	78.5 ± 0.29

^a^The relative mRNA level was estimated from the results of qRT-PCR as shown in Fig. 2A.

^b^Initial biotransformation rates were calculated based on the product concentration, which was measured at t = 0.5 h by GC/MS (Fig. 3). The experiments were carried out in triplicate.

^c^The product yield was calculated based on the substrate depletion and the product concentration, which was determined by GC/MS (Fig. 3). The experiments were carried out in triplicate.
