# Supplementary material for: 3′-UTR engineering to improve soluble expression and fine-tuning of activity of cascade enzymes in Escherichia coli
Source: Sci Rep. 2016 Jul 11;6:29406. doi: 10.1038/srep29406 (PMC4942690; doi:10.1038/srep29406)
Supplement: Supplementary Information [file srep29406-s1.doc]

**Supplementary Information**

**3'-UTR engineering to improve soluble expression and fine-tuning of activity of cascade enzymes in *Escherichia coli***

## Ji-Won Song^1^, Ji-Min Woo^1^, Gyoo Yeol Jung^2^, Uwe T. Bornscheuer^3^, and Jin-Byung Park^1,*^

**^1^**Department of Food Science & Engineering, Ewha Womans University, Seoul 120-750, Republic of Korea

^2^Department of Chemical Engineering and School of Interdisciplinary Bioscience and Bioengineering, Pohang University of Science and Technology (POSTECH), Gyeongbuk 790-784, Republic of Korea

^3^ Institute of Biochemistry, Department of Biotechnology & Enzyme Catalysis, Greifswald University, 17487 Greifswald, Germany

**Table S1. Oligonucleotides used in this study (5'→3')**

| Oligonucleotide | Sequence |
| --- | --- |

| *hilD*_F | 5'-AAG CTT CAT TTT TTG TAT CTG TCA CTT AAG-3' |
| --- | --- |
| *hilD*_R | 5'-CTC GAG AAT AAA ATG CCG GCC TTA ATC C-3' |
| CAT_F | 5'-AAG CTT ATG GAG AAA AAA ATC ACT GGA TAT-3' |
| CAT257_R | 5'-CTC GAG CGC CCC GCC CTG CCA CTC ATC G-3' |
| CAT357_R | 5'-CTC GAG TCC CAT ATC ACC AGC TCA-3' |
| CAT557_R | 5'-CTC GAG TAT GTG TAG AAA CTG CCG-3' |
| CAT_R | 5'-CTC GAG GGC ATC AGC ACC TTG TCG-3' |
| MO16_F | 5'-CAT ATG TCA CAC ACC GAG ACC GCC GCC GA-3' |
| MO16_R | 5'-GGA TCC TTA ATG ATG ATG ATG ATG ATG CGG GCG GCT GAA GGT CAT GGC GTC GTC C-3' |

**Table S2. Strain and Plasmids used in this study.**

| Strain and plasmid | Genotype or description | Reference or source |
| --- | --- | --- |
| *E. coli* BL21(DE3) | F– ompT gal dcm lon hsdSB(rB- mB-) λ(DE3 [lacI lacUV5-T7 gene 1 ind1 sam7 nin5]) | Novagen |

| pCOLADuet-1-ADH | Carrying the alcohol dehydrogenase gene of *M. luteus* | This study |
| --- | --- | --- |
| pACYCDuet-1-ADH | Carrying the alcohol dehydrogenase gene of *M. luteus* | [^1^](#_ENREF_1) |

| pET-BmoF1 | Carrying the BVMO gene of *P. fluorescens* DSM 50106 | [^2^](#_ENREF_2) |
| --- | --- | --- |
| pET-BmoF1-3’UTR*_hilD_* | Carrying the BVMO gene of *P. fluorescens* DSM 50106  and 3’UTR*_hilD_* | This study |

| pET-BmoF1-3’UTR_CAT257_ | Carrying the BVMO gene of *P. fluorescens* DSM 50106 and 257 nucleotiedes of CAT in 3’UTR | This study |
| --- | --- | --- |
| pET-BmoF1-3’UTR_CAT357_ | Carrying the BVMO gene of *P. fluorescens* DSM 50106 and 357 nucleotiedes of CAT in 3’UTR | This study |

| pET-BmoF1-3’UTR_CAT557_ | Carrying the BVMO gene of *P. fluorescens* DSM 50106 and 557 nucleotiedes of CAT in 3’UTR | This study |
| --- | --- | --- |
| pET-BmoF1-3’UTR_CAT_ | Carrying the BVMO gene of *P. fluorescens* DSM 50106  and CAT sequence in 3’UTR | This study |

| pET-MO16 | Carrying the BVMO gene of *R. jostii* RHA1 | [^3^](#_ENREF_3) |
| --- | --- | --- |
| pET-MO16-3’UTR_CAT257_ | Carrying the BVMO gene of  *R. jostii* RHA1and 257 nucleotiedes of CAT in 3’UTR | This study |

| pET-MO16-3’UTR_CAT_ | Carrying the BVMO gene of  *R. jostii* RHA1 and CAT sequence in 3’UTR | This study |
| --- | --- | --- |

**Scheme S1**. The biotransformation pathway of ricinoleic acid. Ricinoleic acid (**1**) is converted into ω-hydroxyundec-9-enoic acid (**4**) and n-heptanoic acid (**5**) in multistep enzyme reactions. Adopted from our previous study [^1^](#_ENREF_1).

**Scheme S2**. The biotransformation pathway of linoleic acid. Linoleic acid (**6**) is converted into ω-hydroxydodec-9-enoic acid (**11**) and n-hexanoic acid (**10**) in multistep enzyme reactions. Adopted from our previous study [^4^](#_ENREF_4).


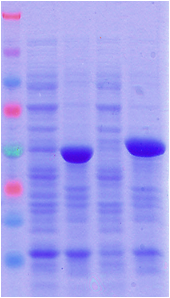


**M**

**S I**

**BmoF1**

**Fig. S1**. SDS-PAGE analysis of cell extracts prepared from culture of the recombinant *E. coli* BL21(DE3) pET22b-BmoF1. Lane M, Marker; Lane S, recombinant *E. coli* BL21(DE3) pET22b-BmoF1 soluble fraction; Lane I, recombinant *E. coli* BL21(DE3) pET22b-BmoF1 insoluble fraction.

A.

CATTTTTTGTATCTGTCACTTAAGTAAAGATTTTTATTAAAATTGTAATAATTTAAAATTCAGACTGCGCATTAACACGCTCTATCAGGATGGGAGGCTATTCAATATCATTGTTCTGTCCGGAAGACAGCTTATACTGATATCTATGGTAATTTAAAGTAAGGCTGATTATATAACACGATTTTTGTGAACTTGTCATCGCTATGATGACTGGTAAAACGATATTGCCTTATTCACAGCGTAAGAATTCGTCCAGATGACACTATCTCCTTCCGGCTTTAACCCTGTGGATTAAGGCCGGCATTTTATT

B.

atggagaaaaaaatcact***ggat***ataccaccgttgat**atat**cccaatggcatcgtaaagaacattttgaggcatttcagtcagttgctcaatgtacctataaccagaccgttcagct***ggat***attacggcctttttaaagaccgtaaagaaaaataagcacaagttttatccggcctttattcacattcttgcccgcctgatgaatgctcatccggagttccgtatggcaatgaaagacggtgagctggtgatatgggaTagtgttcacccttgttacaccgttttccatgagcaaactgaaacgttttcatcgctctggagtgaataccacgacgatttccggcagtttctac***acat***a**tat**tcgcaagatgtggcgtgttacggtgaaaacctggcctatttccctaaagggtttattga***gaat***atgtttttcgtctcagccaatccctgggtgagtttcaccagttttgatttaaacgtggccaatatggacaacttcttcgcccccgttttcactatgggcaa**atat**tatacgcaaggcgacaaggtgctgatgccgctggcgattcaggttcatcatgccgtctgtgatggcttccatgtcggcagaatgcttaatgaattacaacagtactgcgatgagtggcagggcggggcg

C.

**Fig. S2**. (A) Sequence of the *hilD*-3’UTR from *S. enterica*. (B) Coding region of the CAT-657 from plasmid pACYCDuet-1*.* (C) The number of putative RNase E cleavage sites of the 3’UTRCAT variants. The CAT-257, CAT-357, and CAT-557 were indicated by yellow, grey, and dark green, respectively. The potential RNase E cleavage sites are shown in red. The putative RNase E cleavage sites were predicted by using the typical RNase E cleavage sites reported (i.e., -(A/G)N↓AU-), where N identifies any nucleotide, and ↓ indicates the cleavage site [^5^](#_ENREF_31). Also, mfold RNA secondary structure program-based analysis was conducted to identify the RNase E cleavage sites in single-stranded regions [^6^](#_ENREF_32). The number of potential RNase E cleavage sites predicted by both methods was similar each other.

A.

**
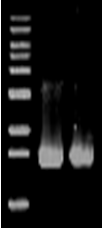
**

**1 2**

***bmoF1***


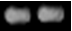


***ihfB***

**1 0.85**

**Relative RNA amount**

B.

**M 1 2 3 4 5**

**
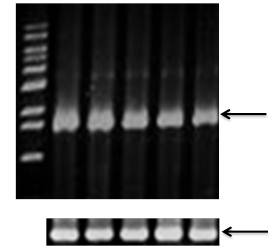
**

**Relative RNA amount**

**1 0.86 0.79 0.54 0.50**

***bmoF1***

***ihfB***

**Fig. S3**. (A) mRNA level of *bmof1-*3’UTR_native_ (lane 1) and *bmof1-*3’UTR*_hilD_* (lane 2) in *E. coli* BL21(DE3). The samples were prepared from cultures of the recombinant *E. coli* BL21(DE3) pET22b-BmoF1-3’UTR_native_ and *E. coli* BL21(DE3) pET22b-BmoF1-3’UTR*_hilD_*. The mRNA level of *ihfB* was used as a control. The relative mRNA level was estimated from the gel electrophoresis by densitometry. (B) mRNA level of *bmof1* with 3’UTR_CAT_ and its variants. The samples were prepared from cultures of the recombinant *E. coli* BL21(DE3) pET22b-BmoF1-3’ UTR_native_ (lane 1), *E. coli* BL21(DE3) pET22b-BmoF1-3’ UTR_CAT-257_ (lane 2), *E. coli* BL21(DE3) pET22b-BmoF1-3’ UTR _CAT-357_ (lane 3), *E. coli* BL21(DE3) pET22b-BmoF1-3’ UTR _CAT-557_ (lane 4), and *E. coli* BL21(DE3) pET22b-BmoF1-3’ UTR _CAT-657_ (lane 5). The mRNA level of *ihfB* was used as a control. The relative mRNA level was estimated from the gel electrophoresis by densitometry.


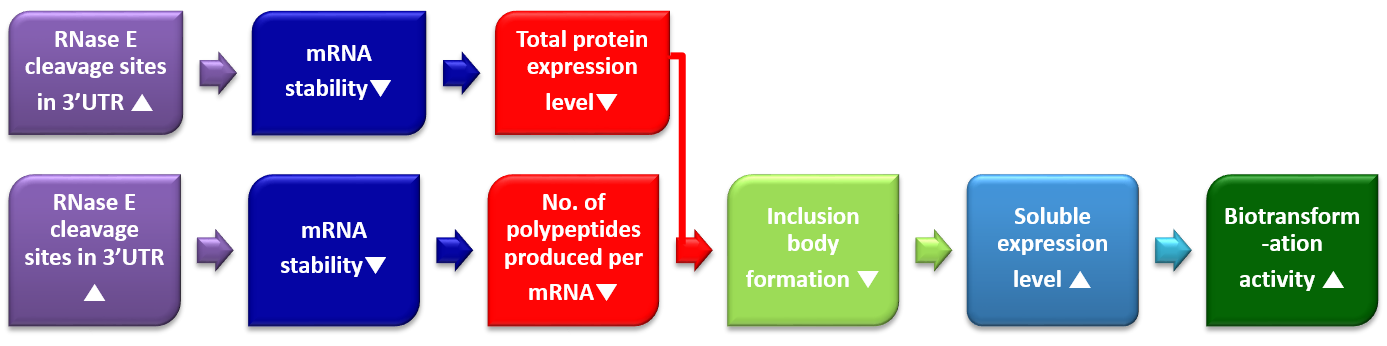


**Fig. S4**. Correlation of the number of RNase E cleavage sites and relative mRNA level, total protein level and soluble protein level. The symbols indicate level of mRNA (●), total protein (●) and soluble protein (●).

A.


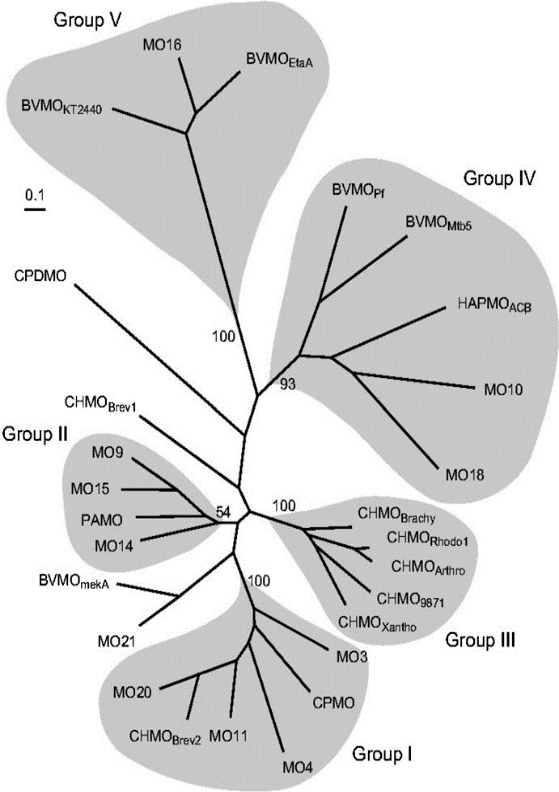


**(BmoF1)**

**Sequence identity : 23.3%**

B.


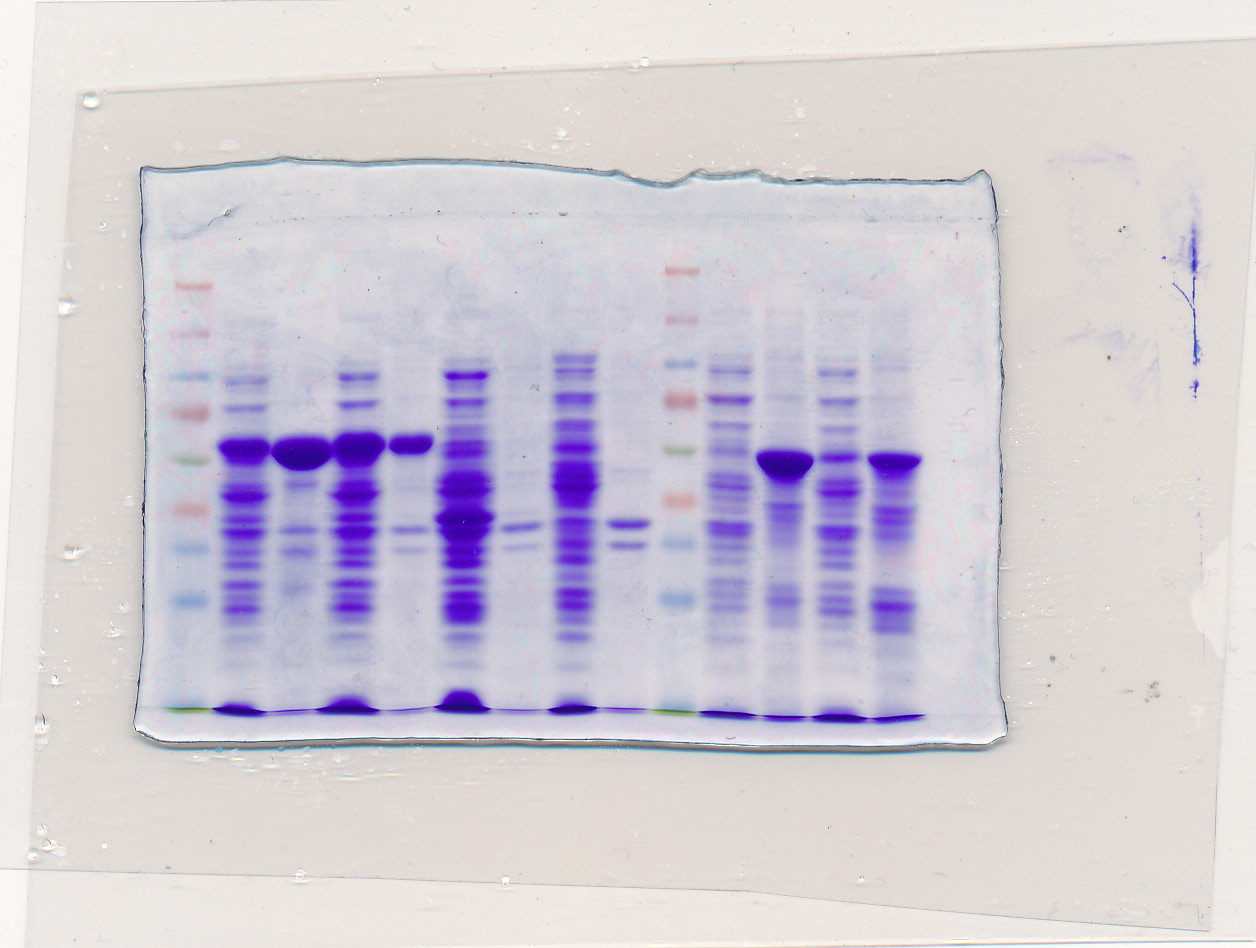

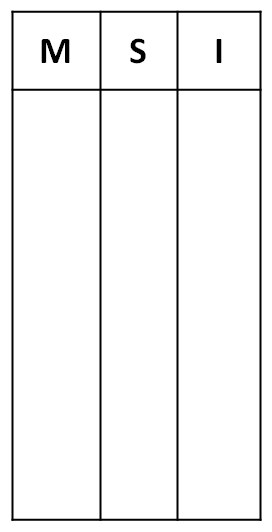


**MO16**

**Fig. S5**. (A) Pylogeny tree of the BVMOs [^3^](#_ENREF_3). (B) SDS-PAGE analysis of cell extracts prepared from culture of the recombinant *E. coli* BL21(DE3) pCOLA-ADH and pET-MO16. Lane M, Marker; Lane S, recombinant *E. coli* BL21(DE3) pCOLA-ADH and pET-MO16 soluble fraction; Lane I, recombinant *E. coli* BL21(DE3) pCOLA-ADH and pET-MO16 insoluble fraction.

A.

B.

C.

**Fig. S6**. Biotransformation of 13-hydroxyoleic acid (7) into ester 9 by (A) the recombinant *E. coli* BL21(DE3) pACYC-ADH, pET-MO16-3’UTR_native_, (B) *E. coli* BL21(DE3) pACYC-ADH, pET-MO16-3’UTR_CAT-257_, and (C) *E. coli* BL21(DE3) pACYC-ADH, pET-MO16-3’UTR_CAT-657_. The experiments were carried out in triplicate and the error bars indicate standard deviation. The symbols indicate concentrations of 13-hydroxyoleic acid (7) (△), 13-ketooleic acid (8) (▼), and ester (9) (■)

**References**

1. Song, J. W. *et al.* Multistep enzymatic synthesis of long-chain alpha,omega-dicarboxylic and omega-hydroxycarboxylic acids from renewable fatty acids and plant oils. *Angew. Chem. Int. Ed.* **52**, 2534-2537 (2013).

2. Kirschner, A., Altenbuchner, J. & Bornscheuer, U. T. Cloning, expression, and characterization of a Baeyer-Villiger monooxygenase from *Pseudomonas fluorescens* DSM 50106 in *E. coli*. *Appl. Microbiol. Biotechnol.* **73**, 1065-1072 (2007).

3. Szolkowy, C., Eltis, L. D., Bruce, N. C. & Grogan, G. Insights into sequence-activity relationships amongst Baeyer-Villiger monooxygenases as revealed by the intragenomic complement of enzymes from *Rhodococcus jostii* RHA1. *Chembiochem* **10**, 1208-1217 (2009).

4. Oh, H. Y. *et al.* Biotransformation of linoleic acid into hydroxy fatty acids and carboxylic acids using a linoleate double bond hydratase as key enzyme. *Adv. Synth. Catal.* **357**, 408-416 (2015).

5. Mackie, G. A. RNase E: at the interface of bacterial RNA processing and decay. *Nature Rev. Microbiol.* **11**, 45-57 (2013).

6. Zuker, M. Mfold web server for nucleic acid folding and hybridization prediction. *Nucleic Acids Res.* **31**, 3406-3415 (2003).
